# High-groove music boosts self-selected running speed and positive mood in female university students

**DOI:** 10.3389/fspor.2025.1586484

**Published:** 2025-04-14

**Authors:** Kazuya Suwabe, Satoshi Kawase

**Affiliations:** ^1^ Faculty of Health and Sport Sciences, Ryutsu Keizai University, Ibaraki, Japan; ^2^ Faculty of Psychology, Kobe Gakuin University, Hyogo, Japan

**Keywords:** groove, physical exercise, exercise adherence, heart rate, affect, self-paced exercise, tempo, sex differences

## Abstract

**Introduction:**

Approximately 27.5% of adults worldwide fail to meet the recommended 150 min per week of moderate-to-vigorous physical activity. Music is a powerful tool that enhances positive affective responses and exercise adherence. However, little is known regarding which types of music are most effective in enhancing these benefits. Groove, which induces a sensation of “wanting to move to the music” and is associated with positive affective responses, may help make self-paced exercise more active and pleasant. Therefore, in this study, we examined the effects of high-groove (HG) music on self-paced exercise and mood responses.

**Methods:**

Thirty-eight university students (18 males) participated in this randomized crossover study under two experimental conditions. The participants ran on a treadmill for 10 min at a self-selected comfortable speed while listening to HG and low-groove (LG) music playlists.

**Results:**

The HG playlist received higher groove ratings than the LG playlist. Self-selected running speed and positive mood responses (vitality and arousal) were greater under the HG condition only in women. Exercise intensity measured using heart rate and the rate of perceived exertion was comparable between the conditions. The groove ratings for the HG playlist positively correlated with speed (HG-LG) and vitality changes under the HG condition (post-pre).

**Conclusion:**

These results suggest that HG music promotes a positive mood response and exercise adherence, particularly in female students. Moreover, the observed sex difference suggests that individual differences in music perception may also influence exercise behavior. Focusing on groove, a musical characteristic distinct from tempo and volume, our study provides a more comprehensive understanding of music that is compatible with exercise, while also examining potential sex differences in its effects on self-paced exercise and mood responses. These findings contribute to health promotion by encouraging active living via physical exercise.

## 1 Introduction

The WHO guidelines for physical activity and sedentary behavior state that physical activity has a positive effect on mental and cognitive health across the human life span and recommend 150 min of moderate-to-vigorous physical activity per week (1). Nonetheless, approximately 27.5% of adults worldwide do not meet this recommendation (2). Previous studies regarding the promotion of exercise adherence have indicated that affective response to physical exercise has been identified as a key factor for enhancing motivation towards exercise and physical activity (3–7). A systematic review of actual surveys concluded that positive affective responses during and after exercise reliably correlated with affective judgments on future physical activity (8). In addition to exercise adherence, positive mood responses to exercise can predict the effects of exercise on mental health (9, 10).

For a more sustainable exercise experience, individualized self-paced exercise prescriptions based on emotional state, rather than at a specific prescribed intensity, have been proposed (11). In affect-based, self-paced exercise, exercise intensity is self-regulated to “feel good” based on affective responses (11, 12). Affect-based exercise not only enhances affective responses but also improves exercise adherence— measured by exercise behavior— particularly in individuals with low physical activity levels and cardiorespiratory fitness (13–15). In addition to the benefits of exercise adherence, exercise training at an affect-regulated intensity also improves aerobic fitness (16). These exercise prescription strategies are also advantageous from the perspective of self-determination theory (17).

Music is one of the most powerful and compatible environmental factors for improving exercise performance and positive mood responses (18, 19). Few studies in the health-promotion literature have examined the effect of music on self-paced exercise at the submaximal level. Edworthy and Waring (20) reported that fast, loud music enhanced exercise intensity and positive mood during treadmill running (20). Hutchinson et al. (21) showed that exercise with self-selected music promoted a greater exercise intensity during 20 min of affect-regulated running, exercise compared to without music while maintaining a “good” feeling (21). Almeida et al. (22) also indicated that fast tempo (140 beats per minute [bpm) music enhances self-selected walking pace in women (22). Although these studies have shown that listening to music increases exercise intensity and positive mood in self-paced exercise, it is unclear whether any musical characteristics other than tempo and volume are effective in enhancing positive affect and exercise behavior.

The groove sensation may be a promising musical feature that enhances affect and exercise behavior (23, 24). A common feature of groove across cultures is “the pleasurable sensation of wanting to move one's body to music” (25–27). Groovy music evokes arousal and pleasant feelings (27, 28). In addition to positive mood responses, groove influences movement. Groove not only affects dance-like movements (29) but also facilitates repetitive and cyclic movements, such as gait and cycling performance. High-groove (HG) music facilitates faster and less variable movements than those performed with low-groove (LG) music (30, 31). Rhythmic entrainment, a fundamental mechanism by which listeners' bodily rhythms adapt to the periodicities of music, may underlie the mechanisms through which groove facilitates movement and elicits a positive mood (32, 33). The coactivation of emotional and motivational brain systems, including the dopaminergic reward pathway, along with the activation of several other areas outside the emotional system, including motor-, attention-, and memory-related regions, is thought to underlie this neural basis (34, 35).

Individual experiences towards groove music are influenced by listener characteristics such as music familiarity (26, 36, 37). Particularly, sex-based differences are known. The tempo at which groove is easily experienced is faster in men than in women (38). In addition, women prefer pop music more (39) and can better identify emotional expressions in music than men (40, 41). These findings suggest sex differences exist in groove sensation, even while listening to the same music.

Taken together, the present study aimed to clarify the effects of groove on self-selected exercise speed and affective response in men and women. We hypothesized that higher groove music would increase self-selected running pace and positive mood compared to lower groove music with a compatible tempo. Furthermore, given that women are generally more sensitive to musical stimuli than men, we anticipated that the observed effects would be more pronounced in women. Our study extends previous research by focusing on groove, a musical characteristic distinct from tempo and volume, while also examining potential sex differences in its effects on self-paced exercise and mood responses.

## 2 Materials and methods

### 2.1 Participants

A priori power analysis was performed using G-power software (42). An optimal total sample size of *N* = 20 for each sex was calculated, with a partial *η*^2^ effect size of 0.17, power of 0.8, and alpha of 0.05, based on our previous acute exercise intervention studies (43, 44).

Forty healthy university students (aged 18–21 years; 20 male participants) from the Faculty of Health and Sports Sciences of Ryutsu Keizai University participated. Those majoring in sports and health sciences were likely more engaged in exercise and had higher physical activity levels than students in non-sports-related fields. Two male participants were excluded because of unaccomplished experiments or missing data, resulting in data from only 38 students (18 male participants) being collected and analyzed. Participants' demographic characteristics are presented in Table 1.

**Table 1:** Participant demographic.

	All		Male		Female	
	Mean	SD	Mean	SD	Mean	SD
Sample size	38		18		20	
Age	19.4	0.8	19.5	0.7	19.4	0.9
Height	165.6	9.6	174.6	6.2	158.2	4.7
Weight	65.5	18.2	79.4	19.0	53.9	5.3
BMI	23.5	4.9	26.1	6.0	21.4	1.8

Written informed consent was obtained from all participants before starting the experiment. Ethical approval for this study was obtained from the Ethics Committee of Ryutsu Keizai University (approval number: 45). This study complied with the ethical requirements of the latest version of the Declaration of Helsinki.

### 2.2 Procedures

**Figure 1:**
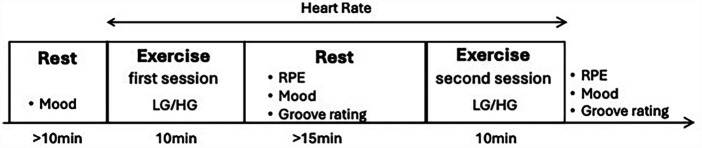
Outline of the experimental procedures. RPE, rating of perceived exertion; HG, high-groove; LG, low-groove

The two experimental conditions, HG and LG conditions, were implemented using a within-subject crossover design in a randomized order. An outline of the experimental procedures is shown in Figure 1. At least 10 min after arrival, the participants performed the first exercise session. The second exercise session was conducted after a minimum of 15 min of seating rest, when the heart rate was confirmed to decrease to resting levels.

As part of the exercise session, the participants ran on the treadmill (PH-OST-2705, Life Fitness) for 10 min, including a 5 min warm-up. The participants were instructed to run, but not walk, for a total of 10 min at a speed that felt comfortable; they were allowed to adjust the treadmill speed by themselves at the beginning and 5 min after the start for 30 s each time. The slope of the treadmill was set to zero. The treadmill speed display was masked from the participants' viewpoint to minimize the impact of cognitive biases. Participants who had never run on a treadmill were allowed to run for 1–2 min before the experiment for familiarization. The first exercise session was then performed after confirming that the heart rate had returned to resting levels following a seated rest. The treadmill speed and heart rate (HR) were recorded continuously during the sessions. HR was measured using a wireless chest strap telemetry system (WHS-1, Union Tool Co., Tokyo, Japan). The rate of perceived exertion [RPE; (45)] was measured immediately after exercise.

### 2.3 Auditory stimuli

We used commercially available music to provide a natural listening experience for the participants (26). The music selection process began with groove ratings for a pool of music tracks, from which a balanced set of high- and low-groove tracks was selected based on these ratings. Although various acoustic features contribute to the perception of groove (27), in this study, we relied on subjective groove evaluations for music selection. First, we selected music that is familiar to Japanese university students since familiarity with a song is known to affect groove (46). Altogether 138 music tracks from a variety of genres (pop, rock, hip-hop, jazz, etc.) were originally listed based on input from lab members from the same faculty as the experimental participants. Nine lab members (six women) rated the groove ratings after listening to music pieces and ranked them. Subsequently, three tracks were selected from the top and bottom of the list to create the HG and LG playlists (Table 2). To minimize the impact of a specific tempo on groove (47) and entrainment (48), music with a variety of tempos (slow, medium, and fast) was included in the playlists. Furthermore, the tempo of the music (bpm) was counterbalanced to be comparable across lists to control the effect of tempo on groove. The bpm were obtained from https://tunebat.com.

**Table 2:** Musical playlist.

No.	Low-groove				High-groove			
	Track	Artist	BPM	Time	Track	Artist	BPM	Time
1	The Sound of Silence	Disturbed	86	4:08	We Will Rock You	Queen	81	2:02
2	Someone You Loved (Lewis Capaldi)	Cynthia Colombo	110	3:14	Butter	BTS	110	2:44
3	Black Swan	BTS	147	3:18	Shake It Off	Taylor Swift	160	3:39

Music was played through loudspeakers, with the volume adjusted to be consistent across all tracks, ensuring it was neither too loud nor too quiet, so as to avoid interference with the sound of the ergometer. The order of the tracks to be listened to in the experiments was randomized across the participants.

### 2.4 Groove rating

We used the Japanese Version of the Experience of Groove Questionnaire (EGQ-JA) to measure the groove experience for each HG and LG music playlists (49, 50). The EGQ-JA consists of six items and evaluates the groove experience using two scales: the urge to move and pleasure. The participants were asked to indicate how they felt when they listened to a piece of music based on a seven-point Likert-type scale ranging from 0 = “strongly disagree” to 6 = “strongly agree”. The total score for the six items was used as the groove rating.

The participants listened to the music tracks again after the exercise sessions and rated each track. The average rating of the three tracks was used as the groove rating for the HG and LG playlists.

### 2.5 Mood scale

The psychological mood state was measured using the Two-Dimensional Mood Scale (TDMS) before and after the exercise session (51). The TDMS is a psychometric scale comprising eight mood-expressing words that describe both pleasurable and arousal states (energetic, lively, lethargic, listless, relaxed, calm, irritated, and nervous). The participants were asked to indicate how they were feeling at the time according to a six-point Likert-type scale ranging from 0 = 'not at all' to 5 = 'extremely'. Vitality level, which represents low arousal–displeasure to high arousal–pleasure (–10 to +10 points), and stability level, which represents high arousal–displeasure to low arousal–pleasure (–10 to +10 points), were measured. Based on these scores, pleasure (vitality + stability: −20 to +20 points) and arousal (vitality – stability: −20 to +20 points) levels were calculated.

### 2.6 Statistical analysis

First, we tested the significance of the differences according to sex and groove experience while listening to the HG and LG playlists. We performed a repeated-measures two-way analysis of variance (ANOVA) of groove ratings by sex and condition, followed by a Bonferroni's *post hoc* test.

Second, we examined the effects of groove on running speed and exercise intensity (HR, RPE) with a paired *t*-test and mood changes with a repeated measures two-way ANOVA. To account for cases wherein groove ratings differed between men and women, an analysis was performed for each sex.

Finally, when there were significant differences in exercise intensity and mood changes between conditions, we examined their relationship with groove experiences.

## 3 Results

### 3.1 Groove ratings

The groove ratings measured using the EGQ-JP are presented in Table 3. A repeated-measures two-way ANOVA by sex (male/female) × music condition (HG/LG) revealed a significant interaction [F(1, 36) = 4.75, *p* = 0.04, partial *η^2^* = 0.12]. A series of *post hoc* comparisons using a Bonferroni test revealed that groove ratings for the HG playlist were significantly greater than those for the LG playlist in both men [F(1, 17) = 36.25, *p* < 0.001, *d* = 1.80] and women [F(1, 19) = 90.60, *p* < 0.001, *d* = 2.82]. However, the groove ratings for the HG playlist were significantly higher in women than in men [F(1, 36) = 10.37, *p* = 0.003, *d* = 1.01]. The results indicating higher groove ratings for HG than those for LG for both men and women confirmed the validity of each playlist. In addition, the groove ratings for the HG playlist in women indicated that women were more appreciative of HG.

**Table 3:** The results of groove ratings, running speed, and exercise intensity.

	Male					Female				
	LG		HG		*P*-value	LG		HG		*P*-value
	mean	SE	mean	SE		mean	SE	mean	SE	
Groove ratings	19.7	1.1	29.0	1.3	<0.001	20.1	1.3	34.0	0.9	<0.001
Running speed 1–5 min (warm-up)	5.0	0.6	5.0	0.5	0.80	5.8	0.6	6.3	0.5	0.14
Running speed 6–10 min	5.9	0.6	6.0	0.6	0.53	6.9	0.7	7.5	0.6	0.05
Heart rate 1–5 min (warm-up)	112.1	4.8	109.6	4.4	0.33	117.2	4.6	119.9	4.2	0.50
Heart rate 6–10 min	121.1	6.4	121.3	5.5	0.94	132.2	6.0	134.4	5.3	0.43
RPE	12.1	0.6	12.7	0.5	0.23	11.0	0.5	11.4	0.4	0.32

### 3.2 Running speed and exercise intensity

The results of running speed and exercise intensity (HR and RPE) are presented in Table 3. A paired *t*-test revealed that running speed (6–10 min) was faster when listening to HG music than LG music in women [*t*(19) = 2.09, *p* = 0.04997, *d* = 0.21]; however, no significant differences were observed in men [*t*(17) = 0.64, *p* = 0.53, *d* = 0.06]. In contrast, running speed at warm-up, RPE, and HR did not differ between the conditions in either men or women (Table 3).

### 3.3 Mood changes

**Figure 2:**
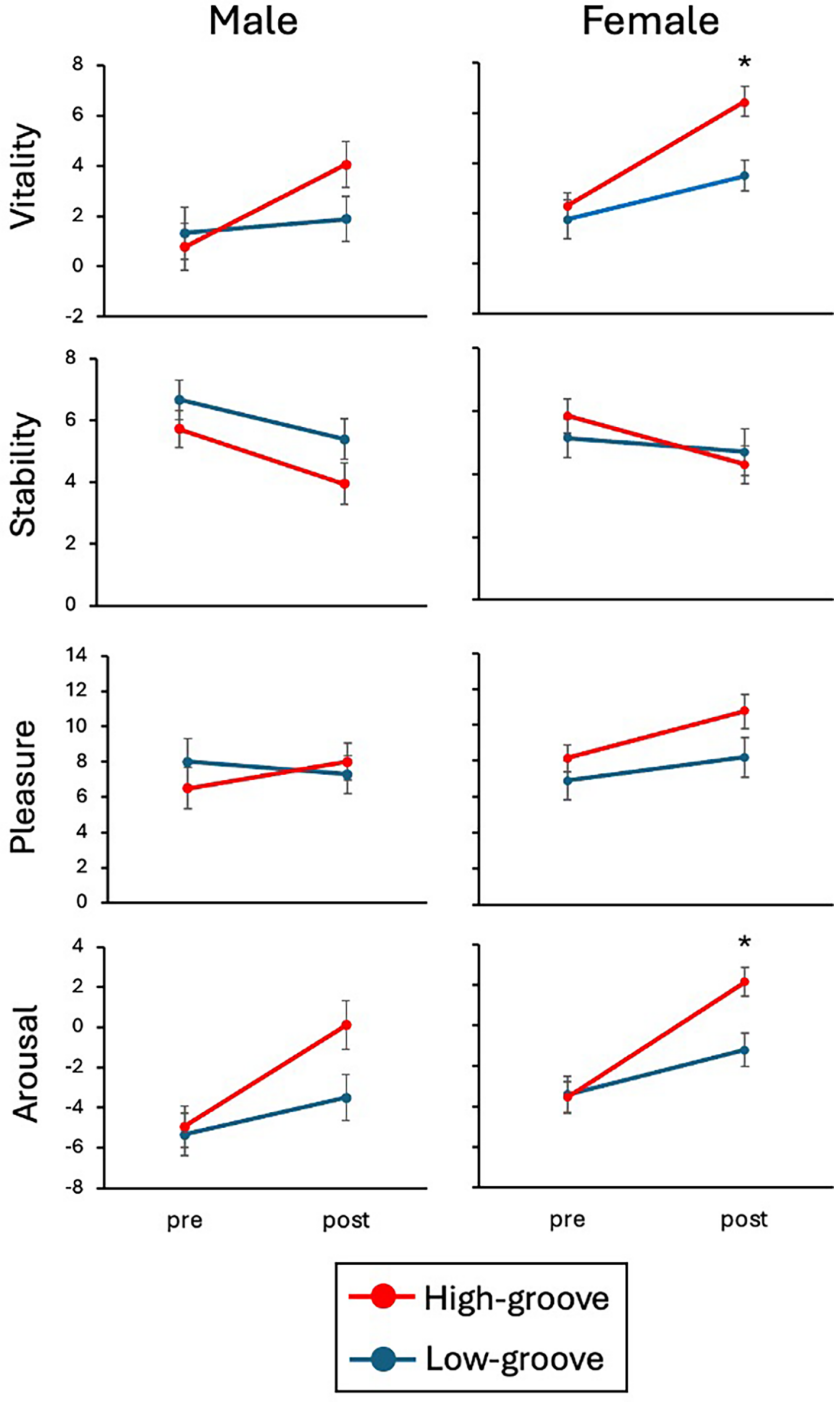
Psychological mood state (vitality, stability, pleasure, arousal) for high- and low-groove conditions in pre- and post-exercise sessions in men and women. Post-exercise vitality and arousal levels were significantly higher under the high-groove condition than under the low-groove condition. * indicates *p* < 0.01 versus low-groove condition after Bonferroni correction.

The psychological mood states (vitality, stability, arousal, and pleasure) measured using the TDMS are shown in Figure 2. In women, a repeated measures two-way ANOVA with experimental condition (LG/HG) and time (pre/post) revealed a significant interaction for vitality [F(1, 19) = 4.69, *p* = 0.04, partial *η^2^* = 0.20] and arousal levels [F(1, 19) = 6.02, *p* = 0.02, partial *η^2^* = 0.24]. A *post hoc* comparison using a Bonferroni test revealed that the vitality [F(1, 19) = 14.44, *p* = 0.001, *d* = 1.67] and arousal levels [F(1, 19) = 11.51, *p* = 0.003, *d* = 1.72] in the post-exercise session for the HG condition were greater than those in the LG condition. There were no interactions for stability levels [F(1, 19) = 2.42, *p* = 0.14, partial *η^2^* = 0.11] and pleasure levels [F(1, 19) = 1.19, *p* = 0.29, partial *η^2^* = 0.06].

In men, there were no significant interactions for validity [F(1, 17) = 2.85, *p* = 0.11, partial *η*^2^ = 0.11], stability [F(1, 17) = 0.32, *p* = 0.58, partial *η^2^* = 0.02], pleasure [F(1, 17) = 1.67, *p* = 0.21, partial *η^2^* = 0.09], and arousal [F(1, 17) = 2.72, *p* = 0.12, partial *η^2^* = 0.14].

### 3.4 Relationship of groove rating to running speed and mood changes

**Figure 3:**
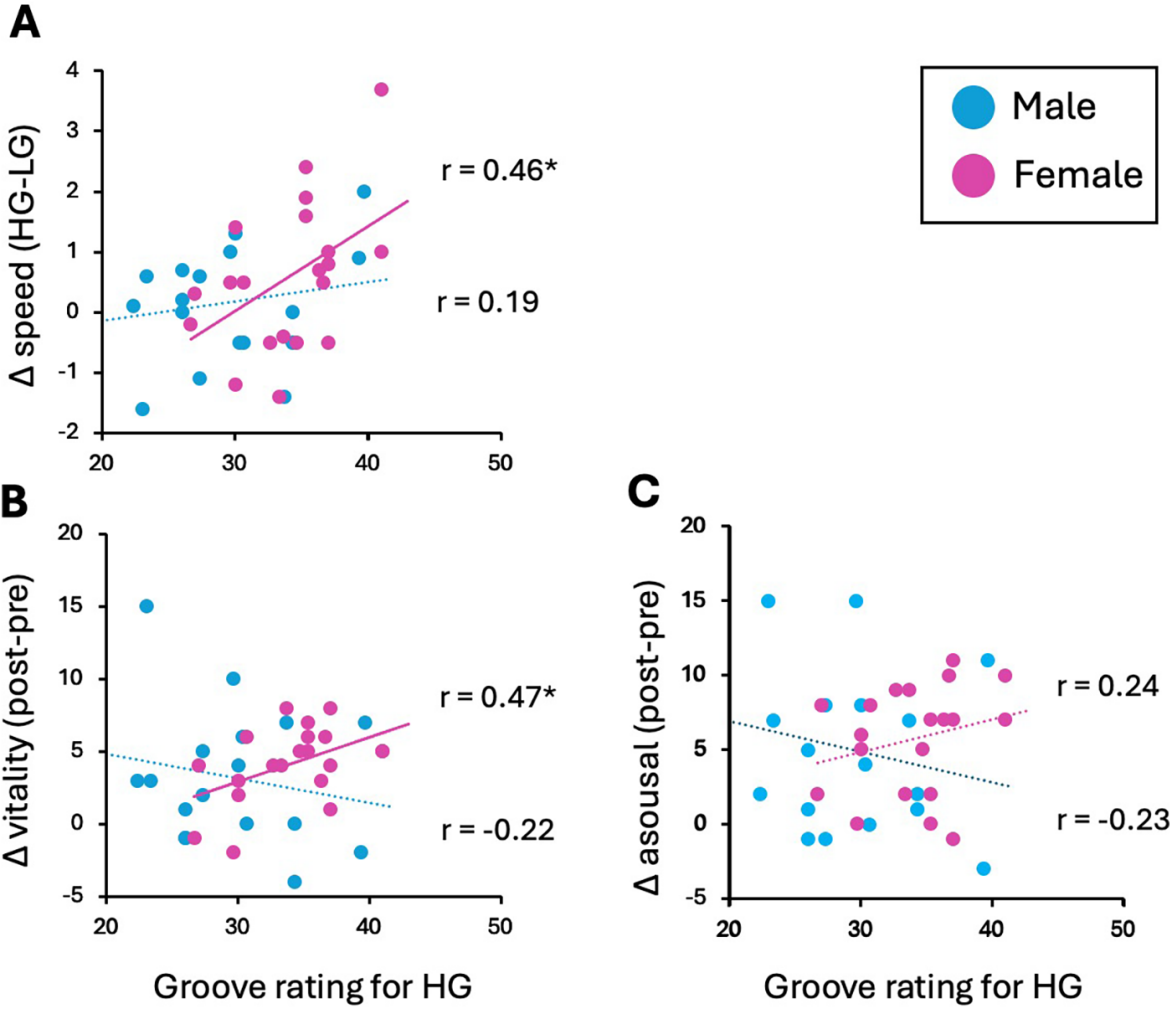
Relationships between groove rating for the high-groove playlist and **(A)** speed change (HG–LG), **(B)** vitality change (post–pre), **(C)** arousal change (post–pre), separately for men and women. Positive correlations were found between groove ratings and both faster running speed and increased vitality in women. r represents Pearson's product-moment correlation coefficient. * indicates *p* < 0.05.

The results of the correlation analyses are shown in Figure 3. In women, the Pearson correlation analysis revealed positive correlations between groove rating in the HG and running speed (HG-LG) (*r* = 0.46, *p* = 0.04) and vitality (post-pre) (*r* = 0.47, *p* = 0.04). There was no significant correlation between the groove rating and arousal (*r* = 0.24, *p* = 0.30). In men, there were no significant correlations between groove ratings and running speed or mood changes.

## 4 Discussion

This study aimed to clarify the effects of HG music on self-selected comfortable running pace and mood responses in male and female university students. The findings in this study revealed that running while listening to HG music induced a higher running speed and more positive mood than that induced by LG music in female students. Furthermore, among female students, the enhanced running speed and vitality levels in the HG condition positively correlated with individual groove ratings for HG music tracks. These findings support our hypothesis, at least in female students, that HG music can make self-paced exercise more active and pleasant and suggest that individual differences in groove experiences may moderate these effects.

Among female students, HG music accelerated running speed and improved positive mood compared to LG music, and these effects were greater in those with higher groove ratings for HG music. These results indicate that groove is involved in the effects of music on running speed and mood, in addition to the previously identified factors such as tempo and volume of music (20, 22). Listening to HG music elicits a faster gait in short-distance measurements (30, 52). The results of this study show that similar effects can be obtained for continuous running. Furthermore, the increased positive mood with HG music is consistent with findings from previous studies which showed that pedaling while listening to HG drum breaks had positive effects on participants' psychological states, including “excited” and “having fun” (53).

Despite the increased running speed in women, exercise intensity (as measured by HR and RPE) remained unchanged. The potential effect of increased exercise intensity due to a higher running speed may have been cancelled out by the effects of HG music on HR and RPE. A previous study has shown that listening to music during exercise can reduce RPE (18). In addition, some reports have indicated that music reduces HR (54, 55). This suggests that, although increased running speed in women may have led to an increase in exercise intensity, the accompanying effects of music on reducing HR and RPE could have counteracted this increase, resulting in no overall change in the perceived exercise intensity.

In the present study, HG music did not affect running speed or mood responses in male participants. There may be two possible explanations for this finding. First, women may be more sensitive to groove than men, and the HG playlist may not have elicited sufficient groove sensations in men. Previous studies suggest that women are more sensitive to the emotional aspects of music than men (40, 41). Additionally, women's affective responses (happy-sad) to music are more influenced by tempo and mode compared to men (56). Since groove is associated with pleasant feelings, women are thought to be more sensitive to groove sensation. Our results, showing that women rated the HG playlist higher than men, support this idea. The biological and neuroscientific mechanisms underlying sex differences remain largely unexplored, thus many aspects are still unclear (57). Second, the contribution of groove as a determinant of running speed and mood responses was less among men. The results of the correlation analysis confirmed that groove rating did not predict the effect on speed and mood in men. Other determinants such as tempo and volume may have a greater impact on men.

This study has some limitations. First, the effect of the synchronization of music and movement is unclear. Since this study did not focus on whether the running steps were synchronized with the music tempo, the running pitch was not recorded. However, the music used in this study ranged between 81 and 159 bpm, whereas the typical running pitch is approximately within 160–200 bpm, suggesting that the music tempo and movement were not synchronized. Music synchronized with movement patterns may further enhance the effect (58). This is a topic for future study. Second, since the participants comprised university students who belonged to the Faculty of Health and Sports Sciences, many were likely familiar with exercise. Additional verification is needed, targeting those with no or fewer exercise habits to promote physically active lifestyles. Third, this study revealed sex differences in the effects of groove on exercise. A more detailed investigation is necessary to determine the factors leading to these individual differences. Applying these findings in practice, considering sex differences is essential to optimize the use of groove music in exercise programs. Fourth, individual differences, including physiological and musical background, as well as music perception, may have influenced the results. Addressing these potential confounding factors in future research could contribute to the development of personalized exercise interventions that account for individual variability. Fifth, the neural basis underlying the observed effects remains unclear. Future studies utilizing neuroimaging methods could provide new insights into the mechanisms through which groove influences exercise performance, as the dopamine reward system and other pathways are likely involved (34, 35).

In conclusion, the present study demonstrated that HG music increased self-selected running speed and positive mood responses compared to LG music, and these effects were greater in female students with higher groove ratings for HG music. These results suggest that HG music promotes a positive mood and exercise adherence, contributing to health promotion through a physically active lifestyle. Coaches and fitness instructors can leverage these benefits by incorporating HG music into exercise programs to enhance motivation and performance. Additionally, integrating HG music into rehabilitation and health promotion programs may help sustain long-term exercise participation.

## Data Availability

The original contributions presented in the study are included in the article, further inquiries can be directed to the corresponding author.
